# Detection of *Mycobacterium angelicum* in Human Urinary Tract, French Polynesia

**DOI:** 10.3201/eid2907.221864

**Published:** 2023-07

**Authors:** Mohamed Lamine Keita, Madjid Morsli, Marc Levy, Grégoire Basse, Cécile Verrier, Michel Drancourt

**Affiliations:** Aix-Marseille University, Marseille, France (M.L. Keita, M. Morsli, M. Drancourt);; Institut Hospitalo-Universitaire Méditerranée Infection, Marseille (M.L. Keita, M. Morsli, M. Drancourt);; Bacterial Virulence and Chronic Infections, INSERM U1047, Department of Microbiology and Hospital Hygiene, CHU Nîmes, University of Montpellier, Nîmes, France (M. Morsli);; Centre Hospitalier de Polynésie Française, Papeete, French Polynesia (M. Levy, G. Basse, C. Verrier)

**Keywords:** *Mycobacterium*, *Mycobacterium angelicum*, whole-genome sequencing, tuberculosis and other mycobacteria, bacteria, zoonoses, French Polynesia

## Abstract

We definitively characterized *Mycobacterium angelicum*, an aquatic zoonotic opportunistic pathogen of the *M. szulgai* complex, using a polyphasic approach that included whole-genome sequencing. The sequence was obtained on the island of Tahiti, French Polynesia, from a urine specimen collected from a patient experiencing a urinary tract infection.

*Mycobacterium angelicum*, a slow-growing mycobacterium associated with animals living in freshwater environments, was delineated within the *M. szulgai* complex by 16S rRNA gene sequencing after its initial isolation from a freshwater angelfish (*Pterophyllum scalare*) in 2003 (*1*). In line with international recommendations (*2*), *M. angelicum* was formally described in 2015 as a new species including the seminal 2003 isolate, 2 additional isolates from freshwater fish, and a fourth isolate recovered from a freshwater tank containing tortoises (*1*). Meanwhile, another isolate was identified in Benin from the rodent *Crocidura olivieri* (*3*). A clinical isolate recovered from a respiratory tract sample taken from a patient in Northern Ireland was also tentatively identified as *M. angelicum* on the basis of 16S rRNA gene sequencing (*4*). We report another clinical isolate identified as *M. angelicum* on the basis of a polyphasic identification approach including whole-genome sequencing (WGS).

A middle-aged patient sought care for active struvite urolithiasis in the left kidney at the main hospital in Papeete, French Polynesia, 18 years after a right nephrectomy for obstructive pyonephrosis. We were unable to follow up with the patient beyond this medical episode. Of 3 successive urine samples collected over 3 consecutive days, which all lacked acid-fast bacilli after Ziehl-Neelsen staining, we successfully cultured 1 urine sample on MGIT (Becton, Dickinson and Company, https://www.bd.com) in 11 days. Culture on Löwenstein Jensen medium (Becton Dickinson) at 37°C under aerobic conditions remained negative after 3 months’ incubation. This isolate positively stained by Ziehl-Neelsen staining; it exhibited rod-shaped, pink-stained bacteria measuring 3.225 + 0.858 μm by 0.717 + 0.048 μm under electron microscopy observation using a SU5000 SEM electron microscope (Hitachi, https://www.hitachi.com). Matrix-assisted laser desorption/ionization time-of-flight mass spectrometry using a Microflex spectrometer and software (Bruker Daltonics, https://www.bruker.com), as previously described (*5*), yielded a noninformative score of 1.31; a derived dendrogram clustered the isolate within the *M. szulgai* group. We conducted WGS concatenating Illumina (https://www.illumina.com) and Nanopore (https://nanoporetech.com) reads using Spades software version 3.15.4, as previously described (*6*); this process yielded 66.4% guanine-cytosine content, 0.23% gap ratio, and a 6,673,592-bp sequence distributed into 51 contigs encoding for 5,707 proteins, 56 tRNA, 3 rRNA, and 2 CRISPRs with a 90.5% total coding ratio. We deposited the sequence into GenBank (submission identification no. 2639860). As a first step, BLAST analysis (https://blast.ncbi.nlm.nih.gov/Blast.cgi) of the 1,326,459-bp longest contig yielded 98% coverage and 99.3% similarity with an environmental *M. angelicum* isolate strain DSM 45057 WGS (GenBank accession no. NZ_MVHE01000100) (*1*) and a 98.6% DNA–DNA hybridization using the Type Strain Genome Server (Leibniz Institute DSMZ, https://www.dsmz.de). As a second step, we used the Orthologous Average Nucleotide Identity tool version 0.93.1 (*7*); the isolate clustered with the best BLAST hit *M. angelicum* isolate with 99.73% genome similarity, whereas further genome sequence similarity values were 93.25% with *M. szulgai*, 93.01% with *M. riyadhense*, and >85% with other mycobacteria (Figure). Those data identified our isolate as the *M. angelicum* Tahiti strain; we deposited it into the Collection de Souches de l’Unité des Rickettsies (CSUR Q5816). No antimicrobial resistance–encoding sequences were predicted in the *M. angelicum* Tahiti strain genome. In agreement with the qualification of *M. angelicum* as a human pathogen by the Center for Genomic Epidemiology online platform (http://www.genomicepidemiology.org), 24 pathogenicity-associated genes were identified in the *M. angelicum* Tahiti strain, all highly conserved in *Mycobacterium* species (Appendix). The location of the *Clp*S gene in 74,165–74,377, its 97.14% sequence similarity with the homologous gene in *Mycobacterium*, and encoding an ATP-dependent Clp protease were predicted as nonpathogenicity factors affecting antimicrobial metabolism and rifampin resistance to protect the *Mycobacterium* cell wall against various stresses (*8*,*9*).

**Figure:**
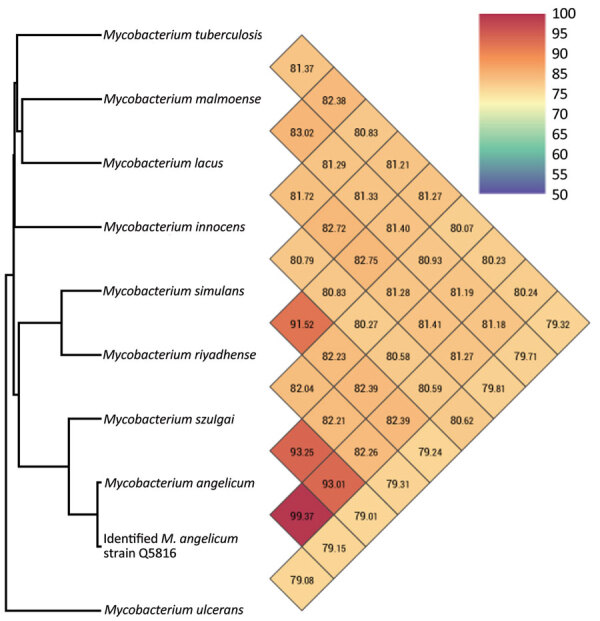
Whole-genome sequence–based clusterization of a *Mycobacterium angelicum* strain from a human urinary tract, French Polynesia. The strain clustered within the *M. szulgai* complex, based on orthoANI calculations (*7*).

We identified the Tahiti strain as *M. angelicum* by combining WGS with phenotypic data in the presence of controls. The identification pathway was an opportunity to make a clinical *M. angelicum* WGS available; 1 *M. angelicum* partial genome sequence (GenBank NZ_MVHE01000100.1) derived from a freshwater angelfish isolate had been described previously (*1*). We did not find reports of *M. angelicum* as a contaminant in that study, in a urine collection device, or as a laboratory contaminant. Furthermore, we did not find previous reports of *M. angelicum* analysis in either of the 2 laboratories that handled this patient’s urine or the strain itself; we concluded that this *M. angelicum* isolate was not a contaminant. This isolate was the only microorganism we were able to isolate by culture from a urine sample; however, its role in the complex urinary tract pathology of this patient remained putative. 

Previously, an isolate of *M. angelicum* was identified from a bronchoalveolar sample collected from a patient with chronic obstructive pulmonary disease in Northern Ireland; identification was based on 100% partial identity (817-bp) 16S rRNA gene sequencing with the reference (*3*). Our study underscores the need for WGS sequencing of bacterial pathogens not identified by first-line phenotypic schemes, including appropriate matrix-assisted laser desorption/ionization time-of-flight mass spectrometry (*5*).

Appendix Additional information about *Mycobacterium angelicum* in human urinary tract.

## References

[1] Pourahmad F, Pate M, Ocepek M, Borroni E, Cabibbe AM, Capitolo E, et al. *Mycobacterium angelicum* sp. nov., a non-chromogenic, slow-growing species isolated from fish and related to *Mycobacterium szulgai.* Int J Syst Evol Microbiol. 2015;65:4724–9. 10.1099/ijsem.0.000642 26420689

[2] Christensen H, Bisgaard M, Frederiksen W, Mutters R, Kuhnert P, Olsen JE. Is characterization of a single isolate sufficient for valid publication of a new genus or species? Proposal to modify recommendation 30b of the Bacteriological Code (1990 Revision). Int J Syst Evol Microbiol. 2001;51:2221–5. 10.1099/00207713-51-6-2221 11760965

[3] Durnez L, Suykerbuyk P, Nicolas V, Barrière P, Verheyen E, Johnson CR, et al. Terrestrial small mammals as reservoirs of *Mycobacterium ulcerans* in benin. Appl Environ Microbiol. 2010;76:4574–7. 10.1128/AEM.00199-10 20435759 PMC2897467

[4] Davies E, Wieboldt J, Stanley T, Maeda Y, Smyth M, Stanley S, et al. Isolation and identification of ‘*Mycobacterium angelicum*’ from a patient with type II respiratory failure: suggested reporting guidelines to molecular clinical laboratories. Br J Biomed Sci. 2012;69:134–6. 10.1080/09674845.2012.12069140 23057162

[5] Robinne S, Saad J, Morsli M, Hamidou ZH, Tazerart F, Drancourt M, et al. Rapid identification of *Mycobacterium tuberculosis* complex using mass spectrometry: a proof of concept. Front Microbiol. 2022;13:753969. 10.3389/fmicb.2022.753969 35432257 PMC9008353

[6] Morsli M, Boudet A, Kerharo Q, Stephan R, Salipante F, Dunyach-Remy C, et al. Real-time metagenomics-based diagnosis of community-acquired meningitis: A prospective series, southern France. EBioMedicine. 2022;84:104247. 10.1016/j.ebiom.2022.104247 36087524 PMC9463524

[7] Lee I, Ouk Kim Y, Park SC, Chun J. OrthoANI: An improved algorithm and software for calculating average nucleotide identity. Int J Syst Evol Microbiol. 2016;66:1100–3. 10.1099/ijsem.0.000760 26585518

[8] Adilijiang G, Feng S, Mi K, Deng H. [Quantitative proteomics analysis of ClpS-mediated rifampicin resistance in *Mycobacterium.*] [in Chinese]. Sheng Wu Gong Cheng Xue Bao. 2014;30:1115–27. 25345012

[9] Marsee JD, Ridings A, Yu T, Miller JM. *Mycobacterium tuberculosis* ClpC1 N-terminal domain is dispensable for adaptor protein-dependent allosteric regulation. Int J Mol Sci. 2018;19:3651. 10.3390/ijms19113651 30463272 PMC6274998

