# Supplementary material for: Detection of Mycobacterium angelicum in Human Urinary Tract, French Polynesia
Source: Emerg Infect Dis. 2023 Jul;29(7):1490–2. doi: 10.3201/eid2907.221864 (PMC10310377; doi:10.3201/eid2907.221864)
Supplement: Appendix — Additional information about Mycobacterium angelicum in human urinary tract. [file 22-1864-Techapp-s1.pdf]

Article DOI: https://doi.org/10.3201/eid2907.221864

EID cannot ensure accessibility for supplementary materials supplied by authors. Readers who have difficulty accessing supplementary content should contact the authors for assistance.

Detection of *Mycobacterium angelicum* in Human Urinary Tract, French Polynesia

Appendix

```
INPUT SEQUENCE INFO:
Input Name ::    2_08_11_2022_090022_149104_input.fsa.translated
Sequences ::      5728
Total bpp ::      2042673
Longest seq ::    6302
Shortest seq ::   30
Avg seq lenght :: 356.0
RESULTS:
Min Identity Threshold ::   91.084
Z-THRESHOLD ::   3.0
Prediction Score ::   100.845
Probability of being human pathogen ::  0.83
Matches::  30
Genome Coverage (%) :: 0.52
Pathogenic Families Matched ::    26
Non-Pathogenic Families Matched :: 4
The organisms is predicted as human pathogenic ::  Yes
MATCHED SEQUENCES:
#input_seq: SEQ4_116 # 132658 # 136821 # 1 #
ID=4_116;partial=00;start_type=GTG;rbs_motif=3Base/5BMM;rbs_spacer=13-
15bp;gc_cont=0.651
#matched_seq:    15642 CP000717   Mycobacterium tuberculosis F11, complete
genome.    Actinobacteridae conserved hypothetical protein    ABR06151
      Yes   92.86
#input_seq: SEQ4_129 # 149255 # 151087 # 1 #
ID=4_129;partial=00;start_type=ATG;rbs_motif=None;rbs_spacer=None;gc_cont=
0.640
#matched_seq:    15642 CP000717   Mycobacterium tuberculosis F11, complete
genome.    Actinobacteridae conserved hypothetical protein    ABR06164
      Yes   93.61
#input_seq: SEQ9_126 # 138779 # 139858 # 1 #
ID=9_126;partial=00;start_type=GTG;rbs_motif=None;rbs_spacer=None;gc_cont=
0.686
#matched_seq:    15642 CP000717   Mycobacterium tuberculosis F11, complete
genome.    Actinobacteridae transcriptional regulatory protein, lacI-
family     ABR07933    Yes   92.2
#input_seq: SEQ21_20 # 15375 # 15761 # -1 #
ID=21_20;partial=00;start_type=ATG;rbs_motif=GGA/GAG/AGG;rbs_spacer=5-
10bp;gc_cont=0.654
```

#matched_seq: 16230 CP000325 Mycobacterium ulcerans Agy99, complete genome. Actinobacteridae conserved hypothetical secreted protein ABL03616 Yes 92.19
#input_seq: Seq1_332 # 390257 # 391426 # 1 # ID=1_332;partial=00;start_type=ATG;rbs_motif=AGxAGG/AGGxGG;rbs_spacer=5-10bp;gc_cont=0.639
#matched_seq: 16230 CP000325 Mycobacterium ulcerans Agy99, complete genome. Actinobacteridae prophage integrase ABL03228 Yes 91.26
#input_seq: SEQ4_125 # 143596 # 144498 # 1 # ID=4_125;partial=00;start_type=ATG;rbs_motif=GGAG/GAGG;rbs_spacer=5-10bp;gc_cont=0.662
#matched_seq: 16725 CP000854 Mycobacterium marinum M, complete genome. Actinobacteridae conserved proteinACC41119 Yes 97.33
#input_seq: Seq1_752 # 856607 # 857344 # -1 # ID=1_752;partial=00;start_type=ATG;rbs_motif=None;rbs_spacer=None;gc_cont=0.646
#matched_seq: 18883 CP000611 Mycobacterium tuberculosis H37Ra, complete genome. Actinobacteridae hexapeptide transferase family protein ABQ74848 Yes 91.84
#input_seq: SEQ5_205 # 256240 # 257085 # -1 # ID=5_205;partial=00;start_type=TTG;rbs_motif=GGAG/GAGG;rbs_spacer=5-10bp;gc_cont=0.656
#matched_seq: 16725 CP000854 Mycobacterium marinum M, complete genome. Actinobacteridae YrbE family protein, YrbE5A ACC42273 Yes 91.81
#input_seq: Seq1_1102 # 1250530 # 1251189 # 1 # ID=1_1102;partial=00;start_type=GTG;rbs_motif=None;rbs_spacer=None;gc_cont=0.626
#matched_seq: 15642 CP000717 Mycobacterium tuberculosis F11, complete genome. Actinobacteridae conserved transmembrane protein ABR07630 Yes 92.24
#input_seq: SEQ4_30 # 42264 # 43037 # 1 # ID=4_30;partial=00;start_type=ATG;rbs_motif=GGAGG;rbs_spacer=5-10bp;gc_cont=0.661
#matched_seq: 88 CP000479 Mycobacterium avium 104, complete genome. Actinobacteridae putative class II aldolase ABK65325 Yes 91.83
#input_seq: SEQ3_550 # 616803 # 617549 # 1 # ID=3_550;partial=00;start_type=ATG;rbs_motif=GGAG/GAGG;rbs_spacer=5-10bp;gc_cont=0.621
#matched_seq: 16725 CP000854 Mycobacterium marinum M, complete genome. Actinobacteridae succinate dehydrogenase (iron-sulfur subunit), Sdh ACC38974 Yes 94.76
#input_seq: SEQ9_127 # 139855 # 140481 # -1 # ID=9_127;partial=00;start_type=ATG;rbs_motif=AGGAG/GGAGG;rbs_spacer=11-12bp;gc_cont=0.652
#matched_seq: 91 AE016958 Mycobacterium avium subsp. paratuberculosis str. k10, complete genome. Actinobacteridae hypothetical protein AAS02808 Yes 94.71
#input_seq: SEQ3_46 # 40867 # 41241 # -1 # ID=3_46;partial=00;start_type=ATG;rbs_motif=GGA/GAG/AGG;rbs_spacer=11-12bp;gc_cont=0.635
#matched_seq: 15762 CP000384 Mycobacterium sp. MCS, complete genome. Actinobacteridae SSU ribosomal protein S12P ABG07098 No 98.39

#input_seq: SEQ4_157 # 180884 # 181501 # 1 #
ID=4_157;partial=00;start_type=GTG;rbs_motif=GGAG/GAGG;rbs_spacer=5-10bp;gc_cont=0.670
#matched_seq: 88 CP000479 Mycobacterium avium 104, complete genome. Actinobacteridae transcriptional regulator, MerR family protein ABK68131 Yes 93.66
#input_seq: Seq1_142 # 175018 # 175335 # -1 #
ID=1_142;partial=00;start_type=GTG;rbs_motif=GGAGG;rbs_spacer=3-4bp;gc_cont=0.651
#matched_seq: 15642 CP000717 Mycobacterium tuberculosis F11, complete genome. Actinobacteridae integration host factor mihF ABR05760 Yes 99.05
#input_seq: Seq1_1079 # 1225443 # 1225802 # 1 #
ID=1_1079;partial=00;start_type=ATG;rbs_motif=GGA/GAG/AGG;rbs_spacer=5-10bp;gc_cont=0.653
#matched_seq: 15642 CP000717 Mycobacterium tuberculosis F11, complete genome. Actinobacteridae conserved hypothetical protein ABR07613 Yes 96.64
#input_seq: Seq1_373 # 429078 # 429446 # -1 #
ID=1_373;partial=00;start_type=ATG;rbs_motif=AGGAG;rbs_spacer=5-10bp;gc_cont=0.653
#matched_seq: 21055 CP001658 Mycobacterium tuberculosis KZN 1435, complete genome. Actinobacteridae conserved hypothetical protein ACT24330 Yes 92.62
#input_seq: SEQ3_542 # 608730 # 609179 # 1 #
ID=3_542;partial=00;start_type=ATG;rbs_motif=None;rbs_spacer=None;gc_cont=0.633
#matched_seq: 16230 CP000325 Mycobacterium ulcerans Agy99, complete genome. Actinobacteridae conserved hypothetical protein ABL03758 Yes 91.95
#input_seq: Seq1_92 # 117518 # 117751 # -1 #
ID=1_92;partial=00;start_type=ATG;rbs_motif=GGA/GAG/AGG;rbs_spacer=5-10bp;gc_cont=0.594
#matched_seq: 18883 CP000611 Mycobacterium tuberculosis H37Ra, complete genome. Actinobacteridae protein-export membrane protein ABQ73192 Yes 96.1
#input_seq: Seq1_378 # 432040 # 432342 # -1 #
ID=1_378;partial=00;start_type=ATG;rbs_motif=GGAG/GAGG;rbs_spacer=5-10bp;gc_cont=0.620
#matched_seq: 15762 CP000384 Mycobacterium sp. MCS, complete genome. Actinobacteridae conserved hypothetical protein ABG08297 No 93.0
#input_seq: SEQ2_224 # 287984 # 288331 # 1 #
ID=2_224;partial=00;start_type=GTG;rbs_motif=GGA/GAG/AGG;rbs_spacer=5-10bp;gc_cont=0.629
#matched_seq: 88 CP000479 Mycobacterium avium 104, complete genome. Actinobacteridae acyl carrier protein ABK65172 Yes 98.26
#input_seq: SEQ9_59 # 64828 # 65124 # -1 #
ID=9_59;partial=00;start_type=ATG;rbs_motif=AGGAG;rbs_spacer=5-10bp;gc_cont=0.640
#matched_seq: 18883 CP000611 Mycobacterium tuberculosis H37Ra, complete genome. Actinobacteridae putative esat-6 like protein ABQ74144 Yes 93.88

#input_seq: SEQ4_122 # 142287 # 142586 # 1 #
ID=4_122;partial=00;start_type=ATG;rbs_motif=GGAG/GAGG;rbs_spacer=5-10bp;gc_cont=0.667
#matched_seq: 16725 CP000854 Mycobacterium marinum M, complete genome. Actinobacteridae PE family protein, PE19 ACC41116 Yes 94.95
#input_seq: SEQ4_123 # 142867 # 143163 # 1 #
ID=4_123;partial=00;start_type=GTG;rbs_motif=AGGAG;rbs_spacer=5-10bp;gc_cont=0.657
#matched_seq: 16725 CP000854 Mycobacterium marinum M, complete genome. Actinobacteridae EsaT-6 like protein Esx ACC43524 Yes 94.9
#input_seq: SEQ6_196 # 221559 # 221843 # -1 #
ID=6_196;partial=00;start_type=ATG;rbs_motif=AGGAG;rbs_spacer=5-10bp;gc_cont=0.660
#matched_seq: 15642 CP000717 Mycobacterium tuberculosis F11, complete genome. Actinobacteridae Esat-6 like protein esxN (Esat-6 like protein 5) ABR06159 Yes 96.81
#input_seq: SEQ6_311 # 348465 # 348749 # 1 #
ID=6_311;partial=00;start_type=ATG;rbs_motif=AGGA;rbs_spacer=5-10bp;gc_cont=0.642
#matched_seq: 16230 CP000325 Mycobacterium ulcerans Agy99, complete genome. Actinobacteridae EsaT-6 like protein EsxN ABL05326 Yes 92.55
#input_seq: SEQ5_52 # 74165 # 74377 # 1 #
ID=5_52;partial=00;start_type=GTG;rbs_motif=None;rbs_spacer=None;gc_cont=0.587
#matched_seq: 15762 CP000384 Mycobacterium sp. MCS, complete genome. Actinobacteridae ATP-dependent Clp protease adaptor protein ClpS ABG09958 No 97.14
#input_seq: SEQ16_63 # 63548 # 63739 # -1 #
ID=16_63;partial=00;start_type=GTG;rbs_motif=AGGAG;rbs_spacer=5-10bp;gc_cont=0.635
#matched_seq: 16230 CP000325 Mycobacterium ulcerans Agy99, complete genome. Actinobacteridae ferredoxin FdxD ABL06117 Yes 93.65
#input_seq: SEQ9_8 # 9472 # 9633 # 1 #
ID=9_8;partial=00;start_type=ATG;rbs_motif=3Base/5BMM;rbs_spacer=13-15bp;gc_cont=0.642
#matched_seq: 15762 CP000384 Mycobacterium sp. MCS, complete genome. Actinobacteridae hypothetical protein ABG10925 No 96.23
#input_seq: SEQ10_21 # 33929 # 34123 # -1 #
ID=10_21;partial=00;start_type=ATG;rbs_motif=GGA/GAG/AGG;rbs_spacer=5-10bp;gc_cont=0.677
#matched_seq: 88 CP000479 Mycobacterium avium 104, complete genome. Actinobacteridae ribosomal protein L35 ABK68440 Yes 92.19
